# Leveraging microcredentials for sustainability literacy in higher education: A case study of reflective thinking and learning impact in science

**DOI:** 10.1371/journal.pone.0351510

**Published:** 2026-07-09

**Authors:** Brittany Lee Vermeulen, Julie M. Old, Michelle C. Moffitt, Jen Dollin

**Affiliations:** 1 Sustainability Education and Partnerships, Western Sydney University, Australia; 2 School of Science, Western Sydney University, Australia; PLOS: Public Library of Science, UNITED KINGDOM OF GREAT BRITAIN AND NORTHERN IRELAND

## Abstract

Microcredentials are becoming increasingly popular in higher education. Despite their growing popularity, there is limited exploration of microcredentials’ potential for lifelong learning and their role in sustainability education within curricula. Furthermore, the use of and evaluation of reflective thinking linked to science and sustainability within these online offerings is unexplored. Undertaken in 2024, a large undergraduate science subject, Complex Case Studies in Science (n = 435), embedded a short microcredential titled ‘Sustain*ability*: Think, Care, Do’ to foster sustainability learning outcomes. The microcredential introduces learners to key concepts of sustainability, understanding diverse worldviews, unpacking their values and how to think systemically, as well as the relevance of these literacies to their science discipline. Our paper explores the learning of a participating cohort of undergraduate students (n = 33) in developing sustainability literacies through this online intervention to culminate in a final reflective assessment of the subject. Using reflexive thematic analysis, we analysed the students’ reflective assessment task. Our findings demonstrate how an online microcredential enhanced science students’ understanding of sustainability, re-evaluation of their daily practices and professional identities, and the development of literacies that grapple with complexity and interdisciplinary solutions across sustainability domains. We also quantified their engagement through behavioural analytics, which varied greatly as expected with an independent self-paced online course. We share these insights as a blueprint for higher education practitioners to integrate online sustainability microcredentials into their curricula at scale.

## Introduction

University graduates are expected to think differently as they explore and investigate the world around them [1,2]. Higher educational institutions are increasingly being tasked (which comes with critique) with the imperative to equip students and graduates from all disciplines to navigate and thrive in a disrupted, complex, and uncertain future [3;4]. Educational responses must focus on developing sustainability literacies, such as values and worldviews, creative, critical, reflective, systems and future thinking capabilities, and enacted through trans- and interdisciplinary approaches [2,3,5–7]. These literacies are essential for imagining and shaping a sustainable future [4,8] and are just as relevant to science. These extend beyond technical competencies of science, requiring individuals to adopt a critically reflexive stance toward both personal development and evolving professional practices [9]. In a complex, ever-changing modern world, science graduates need to be curious global citizens who can understand sustainable development with an ethical, conscious and inclusive perspective [10; 11].

Microcredentials are gaining popularity in higher education as a flexible solution to address the skills gap in the rapidly evolving global job market [12; 13]. These often short, targeted programs offer granular, immediately applicable skills and allow for faster curriculum updates to match industry trends [12]. Their application for delivering on sustainability is diverse, contentious, and limited.

In this case study, we draw on our experience with the microcredential titled Sustain*ability*: Think, Care, Do (STCD). Launched in 2022 on Open Learning, the microcredential was created as an open, short, self-paced, flexible online course that is digitally credentialed. Its design fosters reflective, systems and critical thinking, while incorporating values, worldviews and knowledge for addressing global sustainability challenges. The microcredential introduces learners to key concepts such as the Anthropocene, ecological resilience, and the 2030 Agenda for Sustainable Development, including the Sustainable Development Goals (SDGs) framework. It employs Education for Sustainability (EfS) pedagogies that include participatory, experiential and transformative learning to empower learners to act and contribute to a more sustainable future.

The STCD microcredential was redesigned and adapted for integration into Complex Case Studies in Science, an undergraduate subject mandatory for Bachelor of Science and Bachelor of Medical Science students, offered in the 2024 Spring semester. STCD aims to build learners’ sustainability literacy through a series of well-designed online activities. For this study, STCD was contextualised and condensed into 10-hours of online learning for this subject. Key learning objectives encompass expressing key concepts of sustainability, exploring real-world sustainability initiatives relevant to science, and practising sustainability skills and applying them to science, or their career path and personal life. To meet the desired objectives, the microcredential design required a pedagogy that challenged students to “participate actively, thinking critically and reflect” [9, p.1478]. Specifically, Education for Sustainability was the main pedagogy that informed the design and facilitated learning. We focused on transformative learning, participatory approaches and reflective thinking [14], aligning with pedagogical orientations of the facilitators towards student-centred approaches [15]. In this paper, we present our interpretation of how the microcredential contributed to the student’s ability to think and reflect critically, care deeply, and take meaningful action as articulated in a final reflective assessment that synthesises their learnings with the course. We argue that STCD adds value to science students’ sustainability literacies and professional identity through engaging rigorously with theoretical knowledge applied to their field of practice demonstrated by their final reflective assessment [16]. We seek to address two research questions:

1. How does the online delivery of this microcredential support science student learning for sustainability?
2. How has the microcredential shifted students’ understanding of the business of science and the complexity of real-world challenges?

### Microcredentials, sustainability and higher education

Microcredentials have been strongly associated with the concept of lifelong learning [13], which over the last decade has had an increased focus on competence development and employability [17]. The range of offerings under the microcredential umbrella is vast and includes nano-credentials, digital and open badges, Massive Open Online Courses (MOOCS), certificates and certifications with varying pricing and fee structures [18]. Digital microcredential technologies have rapidly emerged alongside and across the educational landscape, broadly embracing e-learning technology-based approaches to workforce readiness and rapid upskilling [19; 13]. For higher education institutions, it has been argued that microcredentials can be a complementary or additional feature to an academic program [18]. Critiques of microcredentials have been made as offerings that propagate “zombie skills sets’” [16 p. 12] that facilitate gig work. A further criticism is that microcredentials support the loss of transferable learning if they have a focus on skills without relevant context [20]. In Australia, a microcredential is defined as “as a certification of assessed learning or competency, with a minimum volume of learning of one hour and less than an AQF [Australian Qualifications Framework] award qualification, that is additional, alternate, complementary to or a component part of an AQF award qualification” [21]. This definition of a microcredential is particularly important as it sets microcredentials, like STCD, apart from other online formats by ensuring that learning is designed and validated through assessment.

In higher education, the field of research on microcredentials is still ascendant, with academic literature limited [22; 19; 23; 24]. A systematic review of the four main types of microcredentials undertaken by Thi Ngoc Ha et al. [25] assessed research conducted between 2012–2022. This review of 29 selected articles found that approximately 40% of the journal articles examined microcredential effectiveness. A further 30% investigated microcredential learning experiences, and the remaining studies were split between a focus on the value of microcredentials and educator perspectives. The major findings from this deductive analysis of empirical evidence were that microcredentials were in early stages of development in higher education, that they are a worthwhile inclusion in higher education and – despite the name – microcredentials are “no micro task for either students or higher education providers” [(Thi 25, p. 1538)].

Literature regarding their learning outcomes, particularly linked to science and sustainability, is limited [26]. A sweep of current offerings in the Australian HE microcredential space by universities indicates a strong bias towards business and operational-based sustainability skills. These include microcredentials that offer participants the skills to build a strategic environmental and social governance plan (RMIT University), climate and net zero emission strategies for business executives (University of Sydney), urban cooling strategies (Western Sydney University), and urban design for bushfire resilient homes (University of Melbourne). We argue that this approach is not enough for the capabilities needed for an evolving world.

Rapid population growth, increasing industrialisation and the growth of the consumer society, twinned with the dominating free market economy and neoliberalism forces, have culminated in the current cascading ecological, economic and social crises of the 21^st^ century [27]. These sustainability challenges are vexing wicked problems. They have no definitive answer or offer one solution as there are so many diverse perspectives complicated by uncertainty, chaos and complexity [28; 29]. Traditional academic disciplines reduce reality to a selection of discrete parts or silos and omit key interconnections [30] and, while vital, are also inadequate in grasping the interconnected sustainability challenges across social, cultural, ecological and economic domains. Over the last half century, Sustainability Education (SE), Education for Sustainable Development (ESD) and Education for Sustainability (EfS) approaches have evolved as a pedagogy of practice and academic philosophy in response to these crises. Wiek et al. [31] argued almost 15 years ago that transitions toward a more sustainable future are fostered through an increased student awareness and the requirement for a specific set of sustainability competencies and skill.

EfS has a long academic trajectory to equip learners with creative, critical, reflective, systems and future thinking skills. EfS emphasises transformative, interdisciplinary learning, while focusing on values and worldviews guiding students to contribute to a more sustainable and diverse society [2; 32; 5; 33; 3]. Despite the urgency of increasing climate change impacts, rising social and economic inequality, biodiversity loss, water scarcity and pollution, food insecurity, waste and resource management, failures in the embedding and uptake of EfS (and its associated acronyms) have remained patchy in higher education [3]. [34, p. 114] found that: “much sustainable development education in higher education takes place in partial or absolute ignorance of the existence of something called ‘Education for Sustainable Development’”, with [35] Perrson et al. (2023, p. 204) also finding that for academics, the “concept of SD [Sustainable Development] was vague concerning their teaching practice”.

Integrating sustainability into science curricula requires addressing concepts including economic, environmental and technological growth [36]. As such, an interdisciplinary approach to teaching for sustainability to science students is required [37]. Developing sustainability literacies (those shaped and defined by EfS) in science students is vital to assist them in navigating the views presented in media, policy and public opinion [37]. Reflective thinking is one such approach that can aid this.

Reflective thinking is a critical component of education (and our design and assessment of STCD) that fosters deep learning and the development of higher-order cognitive skills, such as critical thinking, creativity and problem solving [38; 39]. Dewey [40, p. 6] defines reflective thinking as the “active, persistent, and careful consideration of any belief or supposed form of knowledge in the light of the grounds that support it, and the further conclusions to which it tends”. In essence, it is deep thinking about content and context. It involves the conscious examination of one’s knowledge, beliefs, and cognitive processes, while recognising the influence of social and contextual factors [41]. Reflective thinking has been found by previous literature to support the development of students’ professional identity and sustainability literacy by reshaping perspectives, encouraging reasoned decision-making, and enhancing self-awareness. Moreover, it can influence students’ values, attitudes, and behaviours in ways that promote sustainability stewardship in their everyday context [52]. As such, reflective thinking is not only a pedagogical tool but a foundational element in preparing lifelong learners. Reflective practice is also a key process for supporting EfS. That is the development of critical thinking skills and a commitment to action [42; 43]. Further, embedding reflective practices in learning environments, such as through microcredentials, has been shown to cultivate lifelong learning and equip students with the capabilities needed to navigate global challenges characterised by complexity [38; 43].

## Materials and methods

### Context and background

#### Institutional context.

Our university has been guided by globally accepted EfS frameworks that outline the key capabilities a sustainability literate graduate should possess. The institution has a long history with transformative learning and sustainability education from the development of agricultural curriculum as a sustainability learning system at the Hawkesbury Agricultural College (one of the amalgamating colleges leading to the establishment of the University), to a Social Ecology program, and more recently to the establishment of a Sustainability Graduate Attribute. The recommendation and support from the Dean of Science, who sat on the institutional *Sustainability and Resilience 2030* Decadal Strategy Steering Committee, was key in setting up this opportunity. Important for successful delivery, however, was the subject coordinators’ (and co-authors of this paper) enthusiastic engagement with the process of connecting the central Portfolio of Learning and Teaching unit and willingness to take a risk and wade into the unknown.

### Microcredential structure and content

Offered through Open Learning, a globally available learning management system, WSU’s Sustain*ability*: Think, Care, Do (STCD) structures student learning across three modules, each divided into a series of pages that scaffold and built concepts:

*What the planet needs from us now: Transformation and change* (3.5 hours of online learning).

This module introduced students to core concepts of sustainability, planetary wellbeing and transformative learning, including climate change, the Anthropocene, and the 2030 Sustainable Development Agenda, while guiding them to explore disciplinary contexts and pathways for personal and societal change through a real-world local case study on urban heat.

*Thinking differently: Worldviews, values and sustainability* (3.5 hours of online learning)

This module introduced students to the role of values and worldviews in shaping understandings of sustainability, guiding them to connect these concepts with their own lives and experiences in making change. This module also included an exploration of Indigenous Australian worldviews and cultural approaches to traditional science.

*Thinking systemically: A connected world* (3 hours of online learning)

This module introduced students to systems thinking approaches, including simple and context systems, while guiding them to practice additional core sustainability thinking skills (critical, reflective, creative, multidisciplinary) through a real-world global case study on the lifecycle of fast fashion.

Curated digital content was designed for self-paced learning, with clear signposting and framing provided. Content includes podcasts, videos, readings, interactive activities (such as knowledge checks, term matches, drag and drops, and flip cards), and real-world examples from proposing solutions to urban heat to researching SDG-related science innovations. Reflective posts with curated prompt questions encouraged deeper thinking, and peer-to-peer engagement was fostered using a comment function to create a sense of community. Reflective thinking was targeted using reflective student posts at the end of major sections. These range from ‘*what does sustainability mean to me’*, asking students to think about why it matters to them and how it relates to their future and community context, to ‘*what worldviews resonate with you’,* reflecting on why it is important to them and how their worldview has shaped and/or changed over time. Facilitators also monitor the posts, providing feedback and guidance where needed.

At the end of the three modules, students completed a multiple-choice quiz made up of 10 questions. The final quiz assessed foundation knowledge of the UN Sustainable Development Goals (SDGs), students’ ability to identify relevant SDG targets against real-world scientific innovations, and to evaluate sustainability skills, considerations and practices within broad scientific and case study contexts.

STCD was opened from Week 3 – Week 9 of the subject’s timetable as an independent assessment task. A 2-hour online lecture in Week 3 set the scene for the SDGs and included a virtual tour of the Open Learning platform to assist students with enrolment and platform features.

### Subject context

Complex Case Studies in Science (NATS3044) is a level three mandatory subject for all students enrolled in the second year of the Bachelor of Science and Bachelor of Medical Science undergraduate degrees at Western Sydney University, Australia. The subject provides students with the opportunity to explore and critically examine “real-life” science-based problems that are not straightforward and may appear at first glance to be simple. Often, the case studies investigated require multidisciplinary approaches and involve additional social, cultural, ethical and economic factors. Case studies covered many aspect of science, from how to treat cancer, tackle climate change, to wildlife care and conservation, and are dependent on the supervisory academics’ discipline and expertise involved in the subject from year to year. Students had free choice as to which case study group they joined. Key learning objectives for the subject involve developing and enhancing student awareness and knowledge of the United Nations Sustainable Development 2030 Agenda, including the SDGs, Indigenous perspectives and sustainability thinking skills. In 2024, the subject was delivered online with 435 students enrolled. Students worked in groups of no more than four students, divided across 16 unique case study topics.

Assessments for the subject involved an independent one page written proposal (15%), 1,000 word report summarising their approach to the case study issue (15%), and a 750 word written final reflection on their learnings in the subject (25%). Group work was assessed via a 10 minute group presentation based on the approach to the case study problem (10%) and a group infographic summarising their approach to the case study (20%). The remaining 15% was allocated to a Professional Task, whereby students completed the 10-hour STCD microcredential, completed the three learning modules and key learning activities and concluded with a short assessed multiple-choice quiz.

Careful attention was paid to scaffolding the learning from the microcredential into all assessments. Given the intention of the subject was to develop science students’ thinking skills (or EfS literacies), the final assessment was based on a reflective task. For this final task, students were asked to examine any assumptions or attitudes they may have had before commencing the subject, and to consider how the completion of their assessments, including STCD, may have affected their approach to problem solving for their specific case study topic. The reflective question posed was “has this led to a new understanding of problem solving and/or a new way of approaching the business of science?”. An additional resource deployed to support students’ development of this skill was the inclusion of the Library’s Study Smart guide to reflective writing and an online session with students on this method.

### Study participants

An open call for participation in this study was issued (17^th^ – 30^th^ March 2025) to the entire cohort (n = 435). A total of 36 students agreed to take part in the study, providing written consent. Of those, 33 students had completed the necessary assessment tasks (i.e., the microcredential and final reflection) to qualify for the study. A student identifier number was assigned to each student to maintain anonymity before analysis (Table 1). Drawing from this subject, participants of this study are current undergraduate students enrolled in a Bachelor of Science or Medical Science program from varying majors and minors and diverse case study groups (Table 1).

**Table 1:** Study participants, including the degree of study and specialisations.

Student Identifier	Degree of study	Major	Minor (if applicable)	Case study topic and group number
1	Bachelor of Science	Biology	Education Studies – Secondary Teaching	Astrophysics (Group 8)
2	Bachelor of Science	Zoology		Ecology (Group 7)
3	Bachelor of Medical Science	Anatomy and Physiology		Pharmacology (Group 11)
4	Bachelor of Science	Applied Physics	Education Studies – Secondary Teaching	Astrophysics (Group 8)
5	Bachelor of Science	Forensic Science		Conservation (Group 18)
6	Bachelor of Science	Chemistry		Conservation (Group 18)
7	Bachelor of Science	Sustainable Environmental Futures	Microbiology	Conservation (Group 18)
8	Bachelor of Science (Dual Degree)	Biology	Philosophy	Medical Science (Group 13)
9	Bachelor of Science	Zoology		Conservation (Group 18)
10	Bachelor of Medical Science	Biomedical Science		Medical Science (Group 13)
11	Bachelor of Science (Dual Degree)	Animal Science		Animal Science (Group 14)
12	Bachelor of Medical Science	Biomedical Science	Infectious Diseases	Biomedical Science (Group 3)
13	Bachelor of Science	Biology	Education Studies – Secondary Teaching	Environmental Health (Group 17)
14	Bachelor of Medical Science	Human Nutrition		Microbiome Science (Group 2)
15	Bachelor of Medical Science	Anatomy and Physiology		Nutrition (Group 15)
16	Bachelor of Science	Biology		Nutrition (Group 15)
17	Bachelor of Advanced Science	Applied Physics	Mathematics	Astrophysics (Group 8)
18	Bachelor of Science	Zoology		Geospatial (Group 9)
19	Bachelor of Science	Zoology		Ecology (Group 7)
20	Bachelor of Science	Agrifood and Human Nutrition		Nutrition (Group 11)
21	Bachelor of Science	Zoology		Animal Science (Group 12)
22	Bachelor of Advanced Medical Science	Anatomy and Physiology		Animal Science (Group 14)
23	Bachelor of Medical Science	Anatomy and Physiology		Geospatial Science (Group 9)
24	Bachelor of Science	Biology		Conservation (Group 18)
25	Bachelor of Medical Science	Biomedical Science	Pharmacology	Ecology (Group 7)
26	Bachelor of Medical Science	Anatomy and Physiology		Biomedical Science (Group 1)
27	Bachelor of Medical Science	Anatomy and Physiology	Infectious Diseases	Nutrition (Group 15)
28	Bachelor of Science	Biology		Conservation (Group 18)
29	Bachelor of Medical Science	Anatomy and Physiology		Geospatial Science (Group 9)
30	Bachelor of Medical Science	Anatomy and Physiology		Environmental Health (Group 17)
31	Bachelor of Science	Biology		Biomedical Science (Group 3)
32	Bachelor of Medical Science	Anatomy and Physiology	Pharmacology	Nutrition (Group 6)
33	Bachelor of Medical Science	Biomedical Science	Microbiology	Animal Science (Group 14)

### Data analysis

To deliver STCD, we aimed to explore the impact of the microcredential on science students in developing these literacies through an in-depth qualitative analysis. In doing so, we undertook a reflexive thematic analysis (RTA) of participating students’ final reflective assessments in the subject. We furthermore quantified participating students’ engagement with the online offering through behavioural analytics.

RTA was selected as the most appropriate approach in this qualitative study as it foregrounds the researcher’s interpretation, subjectivity and reflexivity theme development to uncover deeper meaning, biases and experiences beyond content analysis [44; 45]. Our research team is made up of two sustainability educators – facilitators and creators of the microcredential – and two science academics – the subject coordinators – whose specialty is microbiology and immunology, respectively. All four of us taught a case study group.

Guided by the analytic process of RTA, the Braun and Clark [44] six-phase process was used to develop our findings. The familiarisation phase (phase 1) and an inductive coding phase (phase 2) were undertaken independently by each researcher. In a collaborative and open process, our preliminary codes (phase 2) were discussed using extracted excerpts from the students’ reflective assessments that supported our ideas. These coded excerpts were then further analysed and condensed through further collaborative discussion to generate initial themes (phase 3). A total of 17 initial themes were co-created. Through a shared and ongoing review of the themes and data, we refined and redefined them, collapsing and consolidating where appropriate, until final themes were agreed upon (phases 4 and 5). The findings were then jointly analysed and written up (phase 6), as detailed below.

Concurrently, the behavioural analytics from the Open Learning site were evaluated to observe how each participant interacted with the microcredential. Specifically, we analysed quantitative data to consider the time spent, quiz results, and overall engagement levels with reflective posts and interactions with their peers. Our approach enabled us to examine how the online delivery and pedagogical design of the microcredential facilitated student learning in sustainability.

This study [H13152] has been approved by the Human Research Ethics Committee (HREC) at Western Sydney University, Australia.

## Results and discussion

Overall, we found that all participating students (n = 33) illustrated aspects of sustainability literacy demonstrated through their final reflective assessment task. Insights from students affirm this to varying degrees, from an increase in general knowledge and awareness of sustainability to more transformative moments of understanding and reorientation of scientific practice and identity. While focusing on the outcomes of STCD, and not presented here, we also acknowledge the supporting structures and influence of the subject design. We confirm that reflection, a deep engagement with content and context, was the catalyst to support these pivotal learning moments. Through our analysis, four key themes emerged, with the behavioural analytics included in the first theme.

### Engaging online to facilitate sustainability learning

Sustain*ability*: Think, Care, Do (STCD) was created as an introduction to sustainability and its core concepts, such as *“…the Sustainable Development Goals and their ties to tackling real-world challenges.”* (Student 25). Employing EfS pedagogies, it was designed to allow for self-directed learning at scale “using the language(s) which learners most commonly use” to deliver these core concepts [46, n.p]. While sustainability learning occurred, how our students engaged with the wide range of methods, media, and activities varied greatly. For instance, of the participating study cohort (n=33), quantitative analysis showed that the average time spent was 2 hours and 6 minutes completing the 10-hour microcredential, with the shortest time being 45 minutes and the longest 14 hours (Table 2). This wide variation in time commitments and student engagement with the microcredential highlights the pitfalls of independent online learning, where “we envisage a world where students take responsibility for their own learning” [47, p. 13].

**Table 2:** Summary of behavioural analytics data of study participants from the STCD Open Learning Site.

Behavioural Metric	Average	Minimum	Maximum	Expectation of Course
**Time spent on Course**	2 h 6 min	45 min	14 h 51 min	10 h
Quiz result (%)*	70%	50%	100%	0 - 100%
**No. of comments**	1	0	7	7
**No. of kudos**	4	1	12	N/A
Course complete (%)*	100%	–	–	100%

* indicates assessed items of the subject. N/A = not applicable as no expectation set by the course design.

The online platform of Open Learning was selected and set up to support peer-to-peer online engagement through the comments gallery and to build a self-contained community of practice. A large proportion of participating students made between 0–2 comments (Table 2), although the few contributing more actively found it beneficial “*taking in new perspectives shared within the course content*” (Student 31) and that the use of reflective posts at the end of each module *“helped probe me into reflection…”* (Student 22). The passive interaction with peers through ‘kudos’ (equivalent to a ‘like’ on social media) averaged four per student. Given that there were 11 reflective posts and more than half expected peer-peer interaction, student engagement was considered below expectation (Table 2). Hence, while the platform provided an opportunity for deeper connectivity between individual students, it failed to muster meaningful dialogue in its online form, supporting some critiques of online environments from [48].

**Fig 1:**
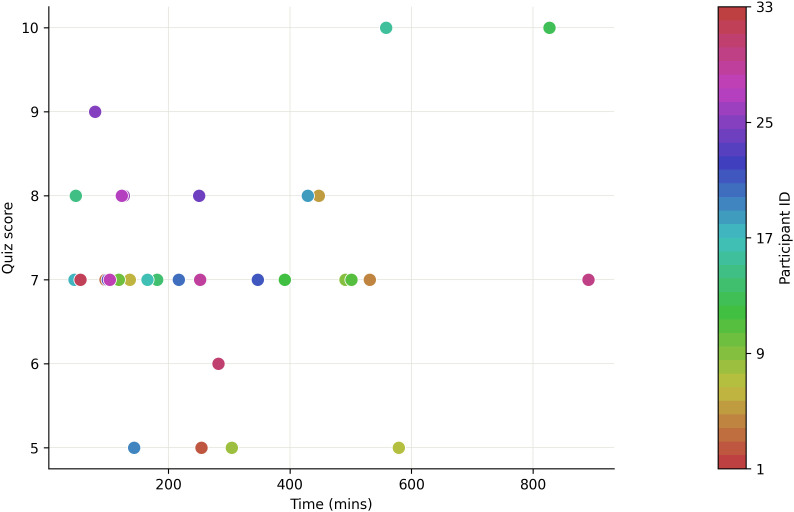
Correlation between total time spent versus quiz score (assessment) by student participantparticipant.

While flexibility is documented to empower self-directed learners, it can lead to further disengagement from others [49]. External and internal motivators for students are recognised as major contributing factors to engagement rates and performance [50]. The assessment design of the subject motivated students to take seriously their reflective posts throughout STCD, as the learning guide made it clear that these could be used in the final assessment. Another motivator was the quiz set up as a completion event for students to gain a digital badge, which was graded. The average quiz result from STCD was 70% (or 7/10), with students scoring between 50% and 100% (Table 2), although time spent did not correlate directly with quiz score (Fig 1). While a multiple-choice quiz provided a measurable knowledge check for the students and facilitators, deeper learning was evidenced through students’ final assessments, structured in a way to apply and reinforce their learning.

Participating students received a digital credential upon completion, as well as academic credit for the subject. Of the total class cohort (n = 435), 398 students enrolled in the microcredential on the Open Learning platform, with 374 students (94%) completing the microcredential and receiving their digital badge. The online modules continued to provide a valuable resource of information for the subject and students’ specific case study topics, for example, *“… when grasping an understanding of how these goals [SDG 3 and 10] are reflected in the inaccessibility of MRI technology.”* (Student 8).

### Professional learning and identity through reflection

The process of reflective thinking was shown to allow students to reshape their professional identity [51]. Professional identity in the sciences is influenced by coursework, authentic research, informal activities, and professional development experiences [52,53]. Through the intervention of STCD, students reported this shift in their identity through their understanding and thinking about enacting science, which was an unexpected outcome:

*At the beginning of this semester, I adopted a rather direct method when it comes to problem-solving and believed that using existing methods and frameworks were the way solutions would be most effectively solved. However, the process of collaborating, integrating feedback, and reflecting on global challenges through the [microcredential] modules significantly broadened my perspective of problem-solving in science.* (Student 33)

*I used to think cosmic rays [case study] were something unrelated [to sustainability], but now I see how important they are... This relates to a comment I made on the module about preserving the environment and advancing long-term well-being. Cosmic rays are inevitable, but they force us to reconsider how we safeguard life on Earth and beyond.* (Student 1)

While not all students achieved the same level of sophistication against the variety of sustainability literacies desired from STCD, many demonstrated an integration into their emerging professional identities and connection to their discipline, with one student reflecting, “*the [microcredential] has been important in showing how scientific inquiry can solve world problems. The most relevant to me has been SDG 3… [it] has direct links to improving health systems around the world.*” (Student 27).

Problem-solving is considered a crucial ‘skill’ in undergraduate science education and of EfS, often linked to its identity, and is thought to be effectively developed by exposing students to authentic, complex problems [54]. However, as challenged by sustainability, there is no singular solution to the multifaceted, complex, messy challenge the students set out to explore in this subject [55]. We found that the act of problem solving gave way to students beginning to grapple with interdisciplinary, holism and perspective-taking [56, 57]. Reflection enabled the students to take on a wider viewpoint whereby they “*… see that problem-solving involves more than just figuring out the most effective answer; it also entails making sure the solution is sustainable and responsible”* (Student 23). Most students, through reflecting on complexity, demonstrated their new and alternate approaches, either in their thinking or the way they applied problem solving to their case study:

*In the beginning, I thought of scientific problem solving as some sort of technical duty you strictly perform in a classroom or lab. But with the [online] modules, I started to understand how scientific solutions are interlinked with larger global problems: inequality, climate change... These reflections significantly impacted my thinking. These modules encouraged me to think beyond the immediate issue and instead about the global context in a holistic approach.* (Student 33)

*I used to think that complex solutions were ineffective because they were economically and logistically challenging. I now understand that it is challenging because it addresses the entire problem rather than isolating a single contributing factor and ignoring other factors. This is what it means to find a sustainable solution... [it] is not always obvious because you may need to account for underlying causes… Thus, desexing dogs and saying it will reduce the stray population [case study topic] is like planting trees and saying it will reverse climate change... Simple measures applied in isolation are meaningless because they do not comprehensively address the underlying causes of a problem*. (Student 22)

Systems thinking was briefly introduced to the students in module 3 of STCD as “thinking systemically in a connected world” and was a taster on complex adaptive systems and the idea of wicked problems. Systemic thinking is thought to motivate science students beyond knowledge acquisition to develop the necessary capabilities to address problems in the context of sustainability [58]. Nuances of systems thinking were reflected on by students through the recognition of the interconnected of sustainability, the SDGs and the world, and their identity as a “systems thinker”:

*One of the most compelling lessons was on thinking systematically within a connected world. This approach broadened my perspective, illustrating the intricate ways systems operate across multiple dimensions simultaneously. As a system thinker, you learn to observe and understand these systems in their entirety before attempting interventions. Applying this new understanding to the [case study] shed light on the complexities in a new way. It reinforced the idea that immediate solutions are not always possible or effective…* (Student 29)

Complementary to the overall subject and STCD learning outcomes, reference to professional development as future scientists were also noted. Students described the microcredential as an important step in their journey to becoming responsible scientists, transforming their research practice. For example, this included that they were *“becoming a better scientist”* (Student 31) and were moving forward *“with excitement to apply this in future scientific work”* (Student 27) while *“highlighting how my own worldview and values influence my approach to science”* (Student 9). Further reflections highlight these tangible outcomes reported by participants:

*The [microcredential], in addition to [other assessment tasks], has allowed a stronger understanding of myself, development of my approach problem solving … and enhancement of multiple professional skills, which I believe has put me on a path to becoming a better scientist.* (Student 31)

*[It] has been enlightening, illustrating the intricate balance between theory and practice in environmental conservation for me because of my passion in this area and … for my future career and endeavours. It has reinforced the importance of adaptability, thorough planning, and the need for continual learning and engagement … to achieve sustainable conservation outcomes.* (Student 28)

### Personal transformation, change and action

EfS emphasises a broad and student-focused nature [56]. The characterisation of a sustainability literate graduate that includes encouraging critical thinking and problem solving, fosters participatory decision-making, and addresses local as well as global issues to name a few [56]. STCD aimed to foster, explore and support students to articulate a wide range of sustainability literacies developed as an outcome of completing the modules. Students alluded to these literacies in their reflections, such as “*expand[ing] my … critical thinking, particularly in the context of global challenges*” (Student 33) and their ability to *“incorporate the concept of interconnectedness … problem solving, with emphasis on collaboration, active communication, empathy and open-mindedness, to achieve holistic solutions that benefit all involved”* (Student 31). This also included action-orientation by *“think[ing] about how I could be a part of global challenges and contribute back with their real-world issues as they make you reflect on the skills that we acquire”* (Student 33).

By empowering students to be active ‘change makers’ in the orientation of STCD, we began to see evidence of how students thought about their personal influence, where *“… in order for sustainability to work, systems and attitudes need to be changed”* (Student 11). A smaller group of students also demonstrated STCD impact on understanding, reflecting and interpreting their values, worldviews and how enacting change involved understanding the perspectives: *“… in the future, I will stress the importance of establishing vision and paradigm to achieve my goals.”* (Student 7). STCD, which included a personal values survey and differentiation between worldview typologies including Indigenous worldviews, was reported to have an impact on their personal development, with one student describing a shift toward a more ‘ecocentric’ worldview, aligning with Lehmann’s [59] emphasis on the role of education in cultivating ecological consciousness:

*The completion of the [microcredential] modules provided the opportunity for personal development regarding my outlook and approaches to sustainability and problem solving. The biggest impact the modules had was to my outlook on my worldview, allowing me to reshape and expand my understandings of the interconnectedness that exist intrinsically through all living beings and the planet. My understanding was developed by taking in new perspectives shared within the course content, such as Indigenous perspectives, as well as considering peer responses on their outlooks and understanding of various worldviews.* (Student 31)

Lighter touchpoints that allude to individual action were also mentioned, including personal habits and behaviours such as *“the importance of preserving and conserving the environment”* (Student 8) and *“helping others in need”* (Student 24). For several students, this was one of their main takeaways in the context of their day-to-day:

*The modules … helped me learn more about how an individual can work to become more sustainable and how as consumers we can change our habits to avoid overconsumption in so many things. [It] also helped me getting a clearer understanding in what the world needs to do as a home to achieve the [SGDs] successfully to ensure we have a sustainable earth for the future.* (Student 21)

*Completing the [microcredential] was a valuable learning experience. The case studies and [reflective] questions helped me understand sustainability, and how our actions impact the world.* (Student 30)

Transformative education in sustainability encourages educators to address student pessimism and foster hopeful, restorative learning environments [60]. We also found that this orientation towards action and a set of capabilities to do so provided hope to students in an uncertain world, demonstrating the capacity for lifelong learning beyond the microcredential:

*This case study and the [microcredential] have made me think more about my role in society and how I can be a more active participant in creating positive change... [and] my own potential to contribute. I began to think more critically about what I could do to help, whether through small actions... I felt inspired by the idea that even individual actions can have a positive impact, especially when aligned with broader global initiatives… The modules have made me think more about my role in society and how I can be a more active participant in creating positive change. Ultimately, this unit has given me confidence in my abilities and a more optimistic outlook on the future”*. (Student 24)

### Global perspectives of science for sustainability and society

A global view of sustainability has been described as a “challenging concept for many students accustomed to the culture of a mono-disciplinary culture” [61, n.p]. Students’ sustainability knowledge, often linked to the environment, particularly from technical disciplines such as science [30], was evidenced and expanded. Student understanding, assumptions, and prior knowledge were challenged, reflected upon and transformed because of STCD:

*I thought I knew everything about sustainability going into this unit. In [Year 12 at high school], I studied sustainability... I was blind about the other [non-environmental] aspects of sustainability until I studied this subject. These were the social aspects like no poverty, gender equality, zero hunger, good health and wellbeing, and partnerships.... These aspects are just as important  ...* (Student 19)

By integrating systems thinking into science education, for example, via the microcredential, we fostered students to overcome the traditional concepts of sustainability, which are limited to just “greening” or “environmentalism” and enabled understanding of the interconnectedness of nature, society, economic and technologies that define real-world sustainability issues [62]:

*… the modules made me further appreciate the bigger context of solving problems. Particular, the modules on sustainable development truly drove home the important place social, environmental, and economic concerns occupy in as far as global challenges are concerned.* (Student 29)

The role of science in society came to the forefront in new ways for the students. We saw this sentiment reflected on across multiple students in the cohort. Students often reported this shift in their thinking and changing mindsets over the semester. For some, the transformational shift was recognising that science is only part of the solution, with one student reflecting:

*In the beginning, I thought of scientific problem-solving as some sort of technical duty you strictly perform in a classroom or lab. But with the [microcredential], I started to understand how scientific solutions are interlinked with larger global problems: inequality, climate change, and sustainability. These reflections significantly impacted my thinking. These modules encouraged me to think beyond the immediate issue and instead about the global context in a holistic approach.* (Student 33)

For others, the relevance of the big picture became clearer. Embedding reflection in STCD seemed to equip students with the capabilities needed to navigate global challenges characterised by complexity [38; 43]. We found our students were able to see and connect with sustainability and its relevance through the practice of reflection. Reflection became important as students began to unpack abstract concepts at a global level. For one student, the reflective posts combined with exploring others was the influencing factor:

*At first, I failed to see the importance of the [microcredential] in relation to the [case study]. I understood there was a connection between goals 13, 14, and 15 and this project. However, I didn’t understand why it was important; “What is this adding to the project?”... Upon starting the learning modules, I realised the SDGs on their own weren’t the main learning outcome... The point of the modules was to collaborate with peers and communicate ideas about sustainability, worldviews, the future, systems thinking, and leverage points. I found myself agreeing and disagreeing with ideas my peers had. I would communicate ideas like my peers in my own posts and reference media that shaped my beliefs. My peers’ ideas also challenged mine.* (Student 7)

There is additional evidence to support that opportunities in these learning environments exist to reflect and engage. Sterling and Thomas [63] expect the development of a sustainability literate student who can contribute to society through an output that is low in the use of energy and raw materials and high in terms of intellectual capital [56]. STCD has the potential to represent this. The content, activities and reflections hosted on the Open Learning platform helped unpack the big picture and cultivate the idea of responsible scientific citizenship and the role of science in society- *“[its] urged me to think about the long-term viability and moral implications of our choices in addition to the short-term fix”* (Student 23). The idea of a globally (inter)connected world was captured by students in supporting their understanding of science in society, something that was transformational and long-term for them as individuals:

*I learned that big problems need sustainable, long-term solutions that are not only scientifically accurate, but also socially responsible and adjusted to different needs. The [microcredential] reinforced this, particularly when addressing sustainability challenges.* (Student 33)

*Personally… I’ve come to view sustainability not in terms of concerns but also as a matter of making thoughtful decisions that contribute positively to society. Our [case study] objective in designing a trial resonates with this belief. We strived to develop a test that does not push the boundaries of science but also upholds ethical standards and has long-lasting benefits.* (Student 26)

Ultimately, STCD provided pivotal, transformative ‘ah-ha’ moments of “sudden realisation, inspiration, insight, recognition, or comprehension” [64, p. 51] where students *“… learnt the value of sustainability*” (Student 11) and *“… focused on everyone doing their part … especially in the long-term and to a global scale”* (Student 16).

## Conclusion

This article has explored how an online microcredential enhanced science students’ understanding of sustainability and demonstrated their development of sustainability literacies. The evidence presented underscores the importance of reflection and reflective thinking in shaping these learning outcomes, supporting previous studies such as Colomer, et al. [51]. Our approach fosters self-directed, student-centred learning and fills critical gaps in disciplinary knowledge for the subject. Overall, our study reveals a nuanced relationship between the pedagogy, structure and content of the STCD, students’ engagement with (or lack of) the online learning environment, and their capacity to articulate deeper, more meaningful insights through reflection in their final assessment tasks. The findings demonstrate that an embedded microcredential – anchored in complex, action-oriented case studies in class – effectively supports sustainability learning and literacies by making global-local interconnections meaningful and equipping students with tools to engage with global frameworks and concepts, such as the SDGs. This suggests that sustainability literacies, those articulated by EfS, can be fostered through scalable, online microcredentials where reflection and reflective thinking is used as the catalyst.

Although we deem the outcomes of STCD a success for the subject and our students (S1 File), there are further considerations by future practitioners who wish to undertake a similar activity:

- Perspectives on EfS emphasise that it is not a matter of memorising facts, but transformation. A multi-modal assessment approach was therefore embedded within the subject to better reflect the complexity of EfS [65]. It is an important consideration to ensure student learning, unlike other forms of online educational offerings. The structure of the final assessment enabled students to demonstrate broader conceptual engagement, reinforcing the importance of reflective thinking. It also developed students’ critical thinking and problem solving, showcasing personal insight in their learning process, as well as broadened the role of science in society.
- In-built reflective activities designed to foster peer-to-peer engagement did not function as intended. In previous iterations of STCD with smaller cohorts, this was successfully facilitated by coordinators. However, with a significantly larger cohort and limited academic time, we were unable to provide the same level of guidance. As a result, students were left to self-manage this component, and the interactive element of the offering was less participatory than anticipated. Workload implications should be considered to support this, as highlighted by Thi Ngoc Ha et al. [25], that microcredentials are “no micro task”.
- Sustainability literacies are inherently difficult to ‘teach’. We acknowledge the challenges in embedding a meaningful understanding of the SDGs, for example, within learning experiences aimed at addressing complex global issues. Navigating an ever-changing world requires resilience, self-awareness, and a suite of sustainability thinking skills that are essential yet elusive in conventional educational frameworks. Reflection is foundational to achieving this. It supports the argument for a multidisciplinary teaching team such as our own.
- The broader subject, Complex Case Studies in Science, provided a foundation to ground STCD, allowing students to contextualise the abstract concepts of sustainability and translate them to disciplinary application, i.e., science and society. Its embeddedness and scaffolding within the subject further enabled deeper engagement with complex case studies, ensuring the microcredential was integrated rather than simply added on or an independent learning activity.

By situating this study in the context of the emerging research on microcredentials, our article contributes to the debate about their ability to foster genuine and lifelong student learning outcomes. Appreciating the limitations of this study, overall, these forms of learning have a positive influence.

### Limitations

While the scope is limited to science and the potential generalisation of our findings, the insights gained were transferable to all other disciplines. This study has several limitations that should be acknowledged. First, the single-year study and the self-selection of participants may limit the representativeness of the broader student cohort and reduce the generalisability of the findings. Additionally, the assessment design and its reflective expectations may have influenced the depth or style of student responses, shaping the data available for analysis. Further, our use of reflexive thematic analysis reflects a methodological choice suited to interpreting subjective experiences; however, its emphasis on researcher interpretation, reflexivity, and meaning‑making may introduce constraints inherent in qualitative inquiry.

### Future research

Future research might explore how similar structured online microcredentials can be designed, incorporated and applied using reflection in other fields, particularly in light of the university’s responsibility to equip our graduates with the necessary literacies (that is, their *ability* to think, care, and do) to navigate a complex world.

## Supporting information

S1 File Final thoughts and reflections (Appendix). (DOCX)
