# Supplementary material for: Leveraging microcredentials for sustainability literacy in higher education: A case study of reflective thinking and learning impact in science
Source: PLoS One. 2026 Jul 9;21(7):e0351510. doi: 10.1371/journal.pone.0351510 (PMC13349291; doi:10.1371/journal.pone.0351510)
Supplement: S1 File — (DOCX) [file pone.0351510.s001.docx]

**Appendix**

**Final thoughts: Reflections as practitioners in the delivery of STCD in CCSS**

To address a research gap, we offer final reflections on our experience delivering the microcredential, highlighting insights and considerations drawn from our teaching practice.

*As academics, our teaching is often confined to the boundaries of our disciplines and undergraduate coursework tends to reflect this. The microcredential allowed us to confidently introduce sustainability concepts beyond our expertise. In this subject, students are encouraged to explore topics outside of their area of specialisation and collaborate with students from other areas. The implementation of the microcredential was essential to ensuring that students creatively incorporate UN SDG values to explore problems in a unique way while moving beyond the basic notions of greening and recycling. It supported a shift towards more equitable and holistic problem solving, broadening their understanding of what sustainability means in scientific contexts. -* Scientist and Subject Coordinator (20 years of teaching experience)

*The microcredential, designed by SE experts, provided students with a digital badge and a unique, flexible, and engaging way to learn about sustainability. STCD successfully helped students connect their academic learning to sustainability and enabled them to develop a more holistic and personal understanding of complex global issues. The microcredential also encouraged science students to reflect on these issues from a fresh perspective, leading to the development of valuable life skills. Although the initial setup was time-consuming, it will be less so in the future. While face-to-face teaching was reduced, peer engagement, though limited, did occur and will be a point of focus in future iterations. -* Scientist and Subject Coordinator (25 years of teaching experience)

*Delivered in workshop mode with 16 diverse case studies and over 400 students, I was curious how STCD would unfold. Early embedding of the microcredential with the School of Science processes revealed niggling resistance from a few science academics, deep in their discipline, yet lacking broader sustainability literacy - why is this needed, and what use is this for science students?! While this phenomenon is well documented (Scott et al., 2012), this was disappointing given the cascading and multiple global crises humans are enmeshed in and the demands for graduates that can deal with uncertainty and complexity. Unconsciously, I assumed similar resistance from students in reductive disciplines. To my delight, it was these students’ reflections that were some of the most powerful, articulate and holistic.* - Sustainability Practitioner and STCD Facilitator (20 years of HE experience)

*Embedding STCD was both exciting and challenging - a first, embedding the offering into a subject at scale, and a test of patience as we navigated independent online learning on a platform outside the usual university systems. I assumed that translating abstract sustainability concepts into disciplinary relevance for science students would expose tensions between technical know-how and EfS (such as criticality, ethics and care). Instead, reflection became a bridge helping students interrogate assumptions and connect personal agency to global impact, within the discipline and beyond. I hadn’t expected this. Their final assessments affirmed for me the value of this offering, which moves beyond techno-scientific solutions to explore values, agency, and societal transformation within science curricula, and the worthiness of this collaboration*. - Sustainability Practitioner and STCD Facilitator (15 years of HE experience)
