# Supplementary material for: Leveraging microcredentials for sustainability literacy in higher education: A case study of reflective thinking and learning impact in science
Source: PLoS One. 2026 Jul 9;21(7):e0351510. doi: 10.1371/journal.pone.0351510 (PMC13349291; doi:10.1371/journal.pone.0351510)
Supplement: Attachment — Submitted filename: Revision Letter.docx [file pone.0351510.s003.docx]

20^th^ March 2026

**Subject: Revision Confirmation for PONE-D-25-60446**

Dear Editorial Team,

Thank you for the opportunity to revise our manuscript, *"Leveraging Microcredentials for Sustainability Literacy in Higher Education: A Case Study of Reflective Thinking and Learning Impact in Science"*. We appreciate the thoughtful and constructive feedback provided by both reviewers and the editorial team.

We have carefully revised the manuscript in accordance with the comments received and is detailed in the table below. A track change version is included to confirm that this work has been undertaken.

## **Point-by-Point Response to Reviewers**

### **Reviewer 1 (Major Revision)**

| **Reviewer Comment** | **Author Response** | **Changes Made** |
| --- | --- | --- |
| Provide additional background information regarding the microcredential program, including pedagogy, structure, and context. | We expanded the background and contextual information describing the microcredentials pedagogical foundations, learning design, and structure. | Introduction and Methods sections expanded to include program pedagogy, structure, and contextual positioning. |
| Include a more detailed description of the microcredential modules, including case studies, reflective assessment, and activities. | We added detailed descriptions of each module, including thematic focus, use of case studies, and reflective learning activities. Where relevant, examples are provided while protecting proprietary materials. | Methods section revised to include detailed module descriptions and reflective assessment structure. Examples included where applicable |
| Clarify how critical reflection skills were developed, including guiding questions. | We clarified how reflective skills were scaffolded through assessment design and explicitly outlined the guiding reflective questions used to stimulate critical reflection. | Methods section updated to include reflective prompts and explanation of reflective skill development. |
| Strengthen the discussion of sustainability literacy skills in the Introduction. | We believe the theoretical framing of sustainability literacy and its relationship to reflective thinking and higher education learning outcomes is strong within this paper. | Introduction revised to explicitly foreground sustainability literacy. |
| “Final Thoughts” should be integrated into results or moved to an appendix. | The “Final Thoughts” section was removed from the main manuscript and relocated to a new Appendix (Supplementary 4). | Conclusion text revised; Appendix created. |
| Correct editorial issue regarding the university name. | The editorial issue has been corrected. | University name corrected in the manuscript. |

### **Reviewer 2 (Minor Revision)**

| **Reviewer Comment** | **Author Response** | **Changes Made** |
| --- | --- | --- |
| “Final Thoughts” section deviates from standard article structure. | The “Final Thoughts” section was removed from the main manuscript and relocated to a new Appendix (Supplementary 4). | Conclusion text revised; Appendix created. |
| Include a dedicated limitations section. | We added a clearly labelled Limitations section to contextualise the findings and scope of the study. | Limitations subsection added, under Conclusion. |

### **Editor and Journal Requirements**

| **Editorial Requirement** | **Author Response** | **Changes Made** |
| --- | --- | --- |
| Ensure manuscript meets PLOS ONE style and file‑naming requirements. | The manuscript was reviewed and revised to align with PLOS ONE formatting and style guidelines. | Formatting and file naming updated. |
| Ethics statement must appear only in the Methods section. | The ethics statement now appears exclusively in the Methods section and has been removed from all other locations. | Ethics statement relocated to Methods. |
| Clarify data availability and any restrictions. | The Data Availability Statement has been revised to explain ethical restrictions on data sharing and includes contact details for data access requests. | Data Availability Statement updated in submission system. |
| Amend authorship list. | Authorship has been corrected in both the manuscript and submission metadata. | Authorship list updated in submission system. |
| Identifying data in the Supporting Information file, S4 Table 3. (Appendix).docx | Supplementary Information (S4) has been removed from the manuscript and as part of the Supporting Information as per journal recommendations. | Removed from submission system. All available data in Results. |
| Identifying data in the Supporting Information file, S5 Statements. (Appendix).docx | Supplementary Information (S5) has been retained; all content has been reviewed and confirmed to contain no identifying or potentially identifiable information. | Appendix created, and Supporting Information file order updated in submission system. |
| Evaluate reviewer‑suggested citations. | Nil suggested publications were provided. | N/A |
| Amendments to manuscript reference list formatting and style. | Reference list has been revised and corrected to Vancouver styling as per guidelines. | Reference list updated and included. |

We hope the revised manuscript now meets the expectations for publication.

Please don’t hesitate to contact me ([b.vermeulen@westernsydney.edu.au](mailto:b.vermeulen@westernsydney.edu.au)) should there be any additional requirements needed.

Sincerely,
Brittany Vermeulen
Corresponding Author
