# Supplementary material for: Leveraging microcredentials for sustainability literacy in higher education: A case study of reflective thinking and learning impact in science
Source: PLoS One. 2026 Jul 9;21(7):e0351510. doi: 10.1371/journal.pone.0351510 (PMC13349291; doi:10.1371/journal.pone.0351510)
Supplement: Peer review history

# Peer review: Decision Letter 0

**DOI:** 10.1371/journal.pone.0351510.r001

**Authors:** Najeh Alsalhi

11 Feb 2026

PONE-D-25-60446

Leveraging Microcredentials for Sustainability Literacy in Higher Education: A Case Study of Reflective Thinking and Learning Impact in Science

PLOS One

Dear Dr. Vermeulen,

Thank you for submitting your manuscript to PLOS ONE. After careful consideration, we feel that it has merit but does not fully meet PLOS ONE’s publication criteria as it currently stands. Therefore, we invite you to submit a revised version of the manuscript that addresses the points raised during the review process.

Please submit your revised manuscript by Mar 28 2026 11:59PM. If you will need more time than this to complete your revisions, please reply to this message or contact the journal office at plosone@plos.org. When you're ready to submit your revision, log on to https://www.editorialmanager.com/pone/ and select the 'Submissions Needing Revision' folder to locate your manuscript file.

Please include the following items when submitting your revised manuscript:

- A letter that responds to each point raised by the academic editor and reviewer(s). You should upload this letter as a separate file labeled 'Response to Reviewers'.
- A marked-up copy of your manuscript that highlights changes made to the original version. You should upload this as a separate file labeled 'Revised Manuscript with Track Changes'.
- An unmarked version of your revised paper without tracked changes. You should upload this as a separate file labeled 'Manuscript'.

If you would like to make changes to your financial disclosure, please include your updated statement in your cover letter. Guidelines for resubmitting your figure files are available below the reviewer comments at the end of this letter.

If applicable, we recommend that you deposit your laboratory protocols in protocols.io to enhance the reproducibility of your results. Protocols.io assigns your protocol its own identifier (DOI) so that it can be cited independently in the future. For instructions see: https://journals.plos.org/plosone/s/submission-guidelines#loc-laboratory-protocols. Additionally, PLOS ONE offers an option for publishing peer-reviewed Lab Protocol articles, which describe protocols hosted on protocols.io. Read more information on sharing protocols at https://plos.org/protocols?utm_medium=editorial-email&utm_source=authorletters&utm_campaign=protocols.

We look forward to receiving your revised manuscript.

Kind regards,

Najeh Rajeh Ibrahim Alsalhi, Ph.D.

Academic Editor

PLOS One

Journal Requirements:

When submitting your revision, we need you to address these additional requirements.

1. Please ensure that your manuscript meets PLOS ONE's style requirements, including those for file naming. The PLOS ONE style templates can be found at

https://journals.plos.org/plosone/s/file?id=wjVg/PLOSOne_formatting_sample_main_body.pdf and https://journals.plos.org/plosone/s/file?id=ba62/PLOSOne_formatting_sample_title_authors_affiliations.pdf

2. Your ethics statement should only appear in the Methods section of your manuscript. If your ethics statement is written in any section besides the Methods, please move it to the Methods section and delete it from any other section. Please ensure that your ethics statement is included in your manuscript, as the ethics statement entered into the online submission form will not be published alongside your manuscript.

3. We note that you have indicated that there are restrictions to data sharing for this study. For studies involving human research participant data or other sensitive data, we encourage authors to share de-identified or anonymized data. However, when data cannot be publicly shared for ethical reasons, we allow authors to make their data sets available upon request. For information on unacceptable data access restrictions, please see http://journals.plos.org/plosone/s/data-availability#loc-unacceptable-data-access-restrictions.

Before we proceed with your manuscript, please address the following prompts:

a) If there are ethical or legal restrictions on sharing a de-identified data set, please explain them in detail (e.g., data contain potentially identifying or sensitive patient information, data are owned by a third-party organization, etc.) and who has imposed them (e.g., a Research Ethics Committee or Institutional Review Board, etc.). Please also provide contact information for a data access committee, ethics committee, or other institutional body to which data requests may be sent.

b) If there are no restrictions, please upload the minimal anonymized data set necessary to replicate your study findings to a stable, public repository and provide us with the relevant URLs, DOIs, or accession numbers. Please see http://www.bmj.com/content/340/bmj.c181.long for guidelines on how to de-identify and prepare clinical data for publication. For a list of recommended repositories, please see https://journals.plos.org/plosone/s/recommended-repositories. You also have the option of uploading the data as Supporting Information files, but we would recommend depositing data directly to a data repository if possible.

Please update your Data Availability statement in the submission form accordingly.

4. Please amend your authorship list in your manuscript file to include author Brittany Vermeulen.

5. Please amend the manuscript submission data (via Edit Submission) to include author Brittany L. Vermeulen.

6. We notice that your supplementary [tables S3] are included in the manuscript file. Please remove them and upload them with the file type 'Supporting Information'. Please ensure that each Supporting Information file has a legend listed in the manuscript after the references list.

7. If the reviewer comments include a recommendation to cite specific previously published works, please review and evaluate these publications to determine whether they are relevant and should be cited. There is no requirement to cite these works unless the editor has indicated otherwise.

Additional Editor Comments :

Dear Authors,

Thank you for submitting your manuscript entitled:

“Leveraging Microcredentials for Sustainability Literacy in Higher Education: A Case Study of Reflective Thinking and Learning Impact in Science”

to PLOS ONE.

The manuscript has now been evaluated by two independent reviewers. Both reviewers find the topic timely and relevant, and they acknowledge the potential contribution of your study to the discussion of sustainability microcredentials in higher education.

However, the reviewers have also raised a number of concerns that need to be addressed before the manuscript can be considered further for publication. Therefore, the editorial decision is:

Major Revision

Please carefully address all comments outlined below and submit a revised version along with a detailed response letter.

Reviewer 1 Comments (Major Revision)

Reviewer 1 finds the manuscript interesting and well written, but requests substantial revisions to strengthen the background, methodology description, and integration of results. Key points include:

Provide additional background information regarding the microcredential program, including clearer details on its pedagogy, structure, and missing contextual elements.

Include a more detailed description of the microcredential modules, such as the case studies adopted, the reflective assessment task, and the reflective activities in each module.

Clarify how critical reflection skills were developed, including specifying the guiding questions used to stimulate students’ reflective thinking.

Strengthen the discussion of sustainability literacy skills in the Introduction.

The section titled “Final Thoughts” should be better integrated into the results analysis rather than appearing as a separate concluding narrative. Alternatively, it may be moved to an appendix.

Please correct the editorial issue noted on page 10 (line 88) regarding the university name.

Reviewer 2 Comments (Minor Revision)

Reviewer 2 considers the study valuable and clearly presented, recommending only minor revisions and editorial refinements. The main suggestions include:

The section “Final Thoughts” should not appear at the end of the manuscript, as it deviates from the typical structure of a research article. The reviewer recommends placing this reflection within the Results section or clearly labeling it accordingly.

The manuscript would benefit from the inclusion of a dedicated limitations section, to help readers better contextualize the findings and interpret any claims of generalizability.

Please submit:

- A revised manuscript addressing all reviewer comments

- A detailed point-by-point response letter explaining how each concern was handled

We look forward to receiving your revised submission.

Sincerely,

Academic Editor

PLOS ONE

[Note: HTML markup is below. Please do not edit.]

Reviewers' comments:

Reviewer's Responses to Questions

**Comments to the Author**

1. Is the manuscript technically sound, and do the data support the conclusions?

The manuscript must describe a technically sound piece of scientific research with data that supports the conclusions. Experiments must have been conducted rigorously, with appropriate controls, replication, and sample sizes. The conclusions must be drawn appropriately based on the data presented.

Reviewer #1: Yes

Reviewer #2: Yes

**********

2. Has the statistical analysis been performed appropriately and rigorously?

Reviewer #1: N/A

Reviewer #2: N/A

**********

3. Have the authors made all data underlying the findings in their manuscript fully available?

The PLOS Data policy requires authors to make all data underlying the findings described in their manuscript fully available without restriction, with rare exception (please refer to the Data Availability Statement in the manuscript PDF file). The data should be provided as part of the manuscript or its supporting information, or deposited to a public repository. For example, in addition to summary statistics, the data points behind means, medians and variance measures should be available. If there are restrictions on publicly sharing data—e.g. participant privacy or use of data from a third party—those must be specified.

Reviewer #1: No

Reviewer #2: Yes

**********

4. Is the manuscript presented in an intelligible fashion and written in standard English?

PLOS ONE does not copyedit accepted manuscripts, so the language in submitted articles must be clear, correct, and unambiguous. Any typographical or grammatical errors should be corrected at revision, so please note any specific errors here.

Reviewer #1: Yes

Reviewer #2: No

**********

5. Review Comments to the Author

Please use the space provided to explain your answers to the questions above. You may also include additional comments for the author, including concerns about dual publication, research ethics, or publication ethics. (Please upload your review as an attachment if it exceeds 20,000 characters)

Reviewer #1: Dear authors,

I would like to congratulate the authors on this interesting article, which assesses the value of a microcredential designed to promote science students’ learning for sustainability and to address the complexity of real-world challenges. Overall, the article is well written and makes a valuable contribution; however, I believe that some important background information regarding the microcredential’s pedagogy, structure, and content is missing. Providing these details in greater depth would strengthen the analysis of the results.

For example, a more detailed description of the microcredential’s pedagogy, content and structure would be helpful, including information about the complex case studies adopted, the final reflective assessment task, and the reflective posts activity in each of the three modules. As a reader and practitioner, I would appreciate a clearer understanding of the specific case studies used and how they were addressed within the microcredential course.

With regard to the development of critical reflection skills, it would be useful to specify which guiding questions were used throughout the microcredential to stimulate students’ reflective thinking. In addition, I would be interested in learning more about the main prior assumptions and understandings held by students that were challenged during the course.

Further discussion of sustainability literacy skills in the introduction would also strengthen the manuscript.

The section “Final Thoughts" on page 33, which presents insights and considerations from practitioners, should be integrated into the results analysis rather than simply reproduced at the end of the manuscript. Alternatively, this section could be included as an appendix.

On page 10, line 88, the name of the university offering the microcredential should be explicitly stated, which is done only later at page 15.

Furthermore, it would be interesting to examine how students’ development of sustainability literacy skills varied according to their major and degree of study.

Reviewer #2: Dear Authors

Thanks for the opportunity to review your paper “Leveraging Microcredentials for Sustainability Literacy in Higher Education: A Case Study of Reflective Thinking and Learning Impact in Science".

This study examines an online sustainability microcredential embedded in a large undergraduate science subject (n=435) in 2024. A cohort of 33 students’ final reflections were analysed using reflexive thematic analysis, with engagement analytics. The microcredential improved sustainability knowledge, systems thinking, and values awareness, prompting shifts in daily practices and professional identity. Results provide a scalable blueprint for integrating sustainability microcredentials in higher education.

The authors have written an interesting and well-structured article that makes a valuable contribution to the discussion of sustainability microcredentials in higher education. The study is clearly presented and offers practical insights for curriculum integration at scale.

Overall, the manuscript requires only minor revisions and a few editorial refinements to further improve clarity and coherence in some sections.

1. The section “Final thoughts: Reflections as practitioners in the delivery of STCD in CCSS” should not appear at the end. In this respect, the manuscript deviates from the typical structure of a research article. I would recommend placing the authors’ own reflection (clearly labelled as such) within the Results section of the article.

2. In addition, the article should include a limitations section to help readers better contextualize and interpret any claims of generalizability.

**********

6. PLOS authors have the option to publish the peer review history of their article (what does this mean?). If published, this will include your full peer review and any attached files.

If you choose “no”, your identity will remain anonymous but your review may still be made public.

**Do you want your identity to be public for this peer review?**  For information about this choice, including consent withdrawal, please see our Privacy Policy.

Reviewer #1: No

Reviewer #2: No

**********

[NOTE: If reviewer comments were submitted as an attachment file, they will be attached to this email and accessible via the submission site. Please log into your account, locate the manuscript record, and check for the action link "View Attachments". If this link does not appear, there are no attachment files.]

To ensure your figures meet our technical requirements, please review our figure guidelines: https://journals.plos.org/plosone/s/figures

You may also use PLOS’s free figure tool, NAAS, to help you prepare publication quality figures: https://journals.plos.org/plosone/s/figures#loc-tools-for-figure-preparation.

NAAS will assess whether your figures meet our technical requirements by comparing each figure against our figure specifications.

# Author comment: Author response to Decision Letter 1

**DOI:** 10.1371/journal.pone.0351510.r002

20 Mar 2026

Thank you for the opportunity to revise our manuscript. We appreciate the thoughtful and constructive feedback provided by both reviewers and the editorial team. We have carefully revised the manuscript in accordance with the comments received. A letter outlining our changes has been provided with further information. We hope the revised manuscript now meets the expectations for publication.

Attachment Submitted filename: Revision Letter.docx

# Peer review: Decision Letter 1

**DOI:** 10.1371/journal.pone.0351510.r003

**Authors:** Najeh Alsalhi, Annesha Sil

29 May 2026

Leveraging Microcredentials for Sustainability Literacy in Higher Education: A Case Study of Reflective Thinking and Learning Impact in Science

PONE-D-25-60446R1

Dear Dr. Vermeulen,

We’re pleased to inform you that your manuscript has been judged scientifically suitable for publication and will be formally accepted for publication once it meets all outstanding technical requirements.

Within one week, you’ll receive an e-mail detailing the required amendments. When these have been addressed, you’ll receive a formal acceptance letter and your manuscript will be scheduled for publication.

An invoice will be generated when your article is formally accepted. Please note, if your institution has a publishing partnership with PLOS and your article meets the relevant criteria, all or part of your publication costs will be covered. Please make sure your user information is up-to-date by logging into Editorial Manager at Editorial Manager® and clicking the ‘Update My Information' link at the top of the page. For questions related to billing, please contact billing support.

If your institution or institutions have a press office, please notify them about your upcoming paper to help maximize its impact. If they’ll be preparing press materials, please inform our press team as soon as possible -- no later than 48 hours after receiving the formal acceptance. Your manuscript will remain under strict press embargo until 2 pm Eastern Time on the date of publication. For more information, please contact onepress@plos.org.

Kind regards,

Annesha Sil, Ph.D.

Staff Editor

PLOS One

Additional Editor Comments (optional):

Reviewers' comments:

Reviewer's Responses to Questions

**Comments to the Author**

1. If the authors have adequately addressed your comments raised in a previous round of review and you feel that this manuscript is now acceptable for publication, you may indicate that here to bypass the “Comments to the Author” section, enter your conflict of interest statement in the “Confidential to Editor” section, and submit your "Accept" recommendation.

Reviewer #1: All comments have been addressed

**********

2. Is the manuscript technically sound, and do the data support the conclusions?

The manuscript must describe a technically sound piece of scientific research with data that supports the conclusions. Experiments must have been conducted rigorously, with appropriate controls, replication, and sample sizes. The conclusions must be drawn appropriately based on the data presented.

Reviewer #1: Yes

**********

3. Has the statistical analysis been performed appropriately and rigorously?

Reviewer #1: N/A

**********

4. Have the authors made all data underlying the findings in their manuscript fully available?

The PLOS Data policy requires authors to make all data underlying the findings described in their manuscript fully available without restriction, with rare exception (please refer to the Data Availability Statement in the manuscript PDF file). The data should be provided as part of the manuscript or its supporting information, or deposited to a public repository. For example, in addition to summary statistics, the data points behind means, medians and variance measures should be available. If there are restrictions on publicly sharing data—e.g. participant privacy or use of data from a third party—those must be specified.

Reviewer #1: Yes

**********

5. Is the manuscript presented in an intelligible fashion and written in standard English?

PLOS ONE does not copyedit accepted manuscripts, so the language in submitted articles must be clear, correct, and unambiguous. Any typographical or grammatical errors should be corrected at revision, so please note any specific errors here.

Reviewer #1: Yes

**********

6. Review Comments to the Author

Please use the space provided to explain your answers to the questions above. You may also include additional comments for the author, including concerns about dual publication, research ethics, or publication ethics. (Please upload your review as an attachment if it exceeds 20,000 characters)

Reviewer #1: The article showed significant improvements compared to the previous version, incorporating the suggestions made in the evaluation process. There is a clearer textual organization, better delimitation of the topic, and improvements in the argumentative structure.

**********

7. PLOS authors have the option to publish the peer review history of their article (what does this mean?). If published, this will include your full peer review and any attached files.

If you choose “no”, your identity will remain anonymous but your review may still be made public.

**Do you want your identity to be public for this peer review?**  For information about this choice, including consent withdrawal, please see our Privacy Policy.

Reviewer #1: No

**********

# Editor report: Acceptance letter

**DOI:** 10.1371/journal.pone.0351510.r004

**Authors:** Najeh Alsalhi, Annesha Sil

PONE-D-25-60446R1

PLOS One

Dear Dr. Vermeulen,

I'm pleased to inform you that your manuscript has been deemed suitable for publication in PLOS One. Congratulations! Your manuscript is now being handed over to our production team.

At this stage, our production department will prepare your paper for publication. This includes ensuring the following:

* All references, tables, and figures are properly cited

* All relevant supporting information is included in the manuscript submission,

* There are no issues that prevent the paper from being properly typeset

You will receive further instructions from the production team, including instructions on how to review your proof when it is ready. Please keep in mind that we are working through a large volume of accepted articles, so please give us a few days to review your paper and let you know the next and final steps.

Lastly, if your institution or institutions have a press office, please let them know about your upcoming paper now to help maximize its impact. If they'll be preparing press materials, please inform our press team within the next 48 hours. Your manuscript will remain under strict press embargo until 2 pm Eastern Time on the date of publication. For more information, please contact onepress@plos.org.

You will receive an invoice from PLOS for your publication fee after your manuscript has reached the completed accept phase. If you receive an email requesting payment before acceptance or for any other service, this may be a phishing scheme. Learn how to identify phishing emails and protect your accounts at https://explore.plos.org/phishing.

If we can help with anything else, please email us at customercare@plos.org.

Thank you for submitting your work to PLOS ONE and supporting open access.

Kind regards,

PLOS ONE Editorial Office Staff

on behalf of

Dr Annesha Sil

Staff Editor

PLOS One
